# Health-Promoting Nutrients and Potential Bioaccessibility of Breads Enriched with Fresh Kale and Spinach

**DOI:** 10.3390/foods11213414

**Published:** 2022-10-28

**Authors:** Marta Czarnowska-Kujawska, Małgorzata Starowicz, Veronika Barišić, Wojciech Kujawski

**Affiliations:** 1 Department of Commodity Science and Food Analysis, The Faculty of Food Sciences, University of Warmia and Mazury in Olsztyn, 10-719 Olsztyn, Poland; 2 Department of Chemistry and Biodynamics of Food, Institute of Animal Reproduction and Food Research of Polish Academy of Sciences, 10-748 Olsztyn, Poland; 3 Department of Food Technologies, Faculty of Food Technology Osijek, Josip Juraj Strossmayer University of Osijek, Franje Kuhača 18, 31000 Osijek, Croatia; 4 Department of Physical Chemistry and Physical Chemistry of Polymers, Faculty of Chemistry, Nicolaus Copernicus University in Toruń, 87-100 Toruń, Poland

**Keywords:** antioxidative capacity, folates, green leafy vegetables, kale, minerals, phenolics, spinach, stimulated digestion

## Abstract

Bread is a staple food and can be a potential product to be enriched with various deficient nutrients. The objective of the study was to characterize the nutritional properties of toasted bread enriched with 10% and 20% of kale and wholemeal bread with 20% and 40% of spinach. The supplementation increased the phenolic content up to 2–3 times in the bread with the addition of 20% spinach and 40% kale. The highest antioxidant properties were noticed in extracts of bread with 20% kale. The *in vitro* digestion released the hydrophilic and lipophilic antioxidative compounds, leading to higher bioaccessibility of the breads enriched with these selected green vegetables. Even more than a 2-fold increase in folate content was observed in breads with the greatest addition of kale (20%) and spinach (40%), from 18.1 to 45.3 µg/100 g and from 37.2 to 83.2 µg/100 g, respectively, compared to the non-enriched breads. Breads with spinach showed significantly (*P* < 0.05) higher contents of all of the tested minerals, Cu, Mn, Fe, Zn, Mg, Ca, Na, K, and P, whereas kale enriched breads showed most of them. The results suggest that the addition of fresh green vegetables can enhance the daily supply of micronutrients and significantly increase the bioavailability of bioactive compounds with high antioxidant status.

## 1. Introduction

According to the World Health Organization (WHO) a healthy-balanced diet including vegetables and fruit helps to protect against malnutrition in all its forms [1]. It is well-known that vegetables are a good source of dietary fiber, antioxidants, phytonutrients, vitamins, polyphenols, and minerals [2]. A vegetable rich diet plays a crucial role in preventing the occurrence of noncommunicable diseases (NCDs) such as diabetes, obesity, cardiovascular disease, and cancer [1,3]. Therefore, any solution contributing to the increase in the daily consumption of vegetables rich in health promoting nutrients [3] is needed. Bread is a staple food and attempts to enrich it with various bioactive ingredients of natural origin have gained popularity in recent years and may lead to an increase to the daily supply of deficient nutrients in the human diet [4,5,6,7,8,9,10].

Kale (*Brassica oleracea* L. var. acephala) contains high levels of bioactive compounds such as glucosinolates, phenolic compounds, and carotenoids [11]. The dominant flavonoids are quercetin and kaempferol, whereas caffeic, ferulic, and sinapic acids are the main phenolic acids of kale. It has been reported that phytochemicals, in synergy with other compounds, are significantly associated with the biological activity of Brassica plants after consumption [12]. Spinach (*Spinacia oleracea* L.) is also a rich source of diverse compounds, whose composition is dominated by carotenoids (e.g., lutein), flavonoid derivatives (e.g., patuletin, spinatoside), phenolic acids (ferulic and *p*-coumaric acids), and lignans [13]. The antioxidant activity of raw spinach and its extracts has been examined in several *in vitro* and in vivo studies [14,15,16,17]. Moreover, it was noted that the application of spinach powder increased the antioxidant activity of durum wheat bread or biscuits [18,19]. To achieve any biological activity, the phytochemicals must be bioavailable, which means that they have to be effectively absorbed from the gut into the circulation system and delivered to the appropriate location within the body [20]. As reported by Saura-Calixto et al. [21], ~48% of consumed polyphenols is digested and potentially absorbed in the small intestine, then in the large intestine, the bioavailability of bioactives is estimated at the level of ~42%. However, ~10% of polyphenols is still not released from the food matrix during the digestion process. Due to this fact, gut microflora could potentially increase the bioaccessibility of polyphenols and therefore antioxidant activity after digestion in the gastrointestinal system. 

Green leafy vegetables are also a rich natural sources of minerals such as potassium, calcium, phosphorus, and magnesium [22,23,24], which have various functions in the body, being building materials for the bones, teeth, skin and hair, maintaining the acid–base balance, having fundamental importance for metabolic processes, and regulating water and electrolyte balance [25]. Kale and spinach are also good sources of folates—vitamin B, which play key roles in providing one carbon unit for nucleotide synthesis and repair as well as vitamins and amino acid synthesis [26,27]. Unfortunately, their deficiency in the diet is common [28]. Meanwhile, low folate status is associated with a higher risk of neural tube defects (NTDs) such as anencephaly and spina bifida in the fetus [29], but also cardiovascular disease, cancer [30], and neurodevelopmental disorders [31]. 

The main objective of the present study was to characterize toasted bread enriched with kale and wholemeal bread enriched with spinach in terms of the selected health-promoting nutrient content. Therefore, the level of folates and minerals as well as the total phenolic compounds and antioxidant ability of extracts were determined. Moreover, the enzymatic digestion *in vitro* of breads was established to observe the changes in the polyphenol contents and their antioxidant activity after stimulated digestion.

## 2. Materials and Methods

### 2.1. Bread Preparation

The bread ingredients (Table 1) used in the present work including fresh spinach and kale were purchased at the local store. Fresh spinach and kale after weighing were blended in a high-speed vacuum blender (Philips HR3752/00, München, Germany) and in this form were added to other ingredients. The breads were prepared and baked in an automatic home baking device (Tefal PF6118 bread maker 1600W, Is-sur-Tille, France). The baking program was selected for wholemeal and toasted bread. For each type of bread, three breads were baked on the same day. After cooling in darkness, the breads were ground in a high-speed vacuum blender (Philips HR3752/00, München, Germany) and forwarded for folate and mineral analysis within the next 2–4 h. Prior to the preparation of extracts for total phenolic content (TPC) and antioxidant activity analysis, the bread samples were lyophilized and ground.

### 2.2. Chemicals and Reagents

Chemicals and reagents (Folin–Ciocalteu phenol reagent, gallic acid, methanol, Na_2_CO_3_) used in the method of total phenolic content and in the *in vitro* digestion (α-amylase from human saliva, Type XIII-A; pepsin from porcine gastrin mucosa, P7000; pancreatin from porcine pancreas 8x USP spec., P7545; and bile extract porcine, B8631) were purchased from Sigma-Aldrich (St. Louis, MO, USA). The Photochem^®^ (Leipzig, Germany) Kit for water-soluble (ACW) and lipid-soluble antioxidants (ACL) was purchased from Analytik Jena (Jena, Germany).

Hydrated lanthanum chloride (Cl_3_La • 7H_2_O), used in mineral content determination, was purchased from Merck (Darmstadt, Germany). Other reagents, ammonium molybdate VI, sodium sulfate IV, and hydroquinone were purchased from “POCH” S. A. (Gliwice, Poland). Standards of the selected minerals, magnesium, potassium, calcium and phosphorus, were diluted with 0.1 M nitric acid at the concentration of 1 mg/cm^3^.

Individual folate standards of folic acid, 5-methyltetrahydrofolate (5-CH_3_-H_4_folate), 5-formyltetrahydrofolate (5-HCO-H_4_folate), and tetrahydrofolate (H_4_folate) were obtained from Sigma Aldrich (St. Louis, MO, USA); whereas 10-formyl folic acid (10-HCO-folic acid) and 5,10-methenyltetrahydrofolate (5,10-CH^+^-H_4_folate) were obtained from Schircks Laboratories (Jona, Switzerland). All standards for folate analysis were prepared as described previously [32]. Chemicals used in the experiments were at least of analytical grade.

### 2.3. Preparation of Bread Extracts and In Vitro Digestion of Samples

The extracts of the control breads and breads enriched with kale and spinach were prepared according to the methodology used previously by Krupa-Kozak et al. [33]. *In vitro* digestion of breads was conducted following the procedure presented by Delgado-Andrade et al. [34] and Bączek et al. [35]. The TPC and antioxidant capacity of the soluble fraction, obtained after the centrifugation of the digested bread samples, were analyzed directly using the described methods (without an extraction step).

### 2.4. Total Phenolic Content (TPC) and Antioxidant Properties of Bread Extracts and Digested Samples Measured by PHOTOCHEM^®^

The TPC method was applied as previously described in details by Krupa-Kozak et al. [33]. The microplate reader (Thermo Scientific, Multiscan^TM^ microplate reader, Altrincham, UK) was used to read absorbance at λ = 755 nm on 96-well plates. Gallic acid (stock solution of 1 mg/mL) was used to build a calibration curve (R^2^ = 0.999) and therefore, the results were expressed as mg/g of dry weight.

The Photochem^®^ apparatus (Analytik Jena, Leipzig, Germany) was used to analyze the ability of bread extracts to scavenge the superoxide anion radical (O_2_^•−^) by water- and lipid-soluble antioxidants (ACW and ACL modes). The photochemiluminescence (PCL) assay was performed as described by Zieliński et al. [36]. Trolox was used as a standard (R^2^ = 0.9988 in ACW; R^2^ = 0.9926 in ACL). The results were calculated as µmol Trolox eq./g of dry weight.

### 2.5. Mineral Determination

Bread samples were weighed in borosilicate glass tubes, and then mineralized in a mixture of nitric and perchloric acids 3:1 (*v*:*v*). The mineralization was carried out in an electric aluminum heating block (VELP DK 20, Scientifica, East Sussex, United Kingdom), and the temperature was gradually increased from 100 to 180 °C in a few hours. The colorless mineralizate obtained was transferred to a 50 cm^3^ volumetric flask and made up with deionized water up to the mark. Reagent samples were prepared in parallel with the test samples.

The determination of copper (Cu), manganese (Mn), iron (Fe), zinc (Zn), magnesium (Mg), and calcium (Ca) was carried out by the flame atomic absorption spectrometry (acetylene–air flame) technique using a Thermo iCE 3000 Series (Madison, WI, USA) atomic absorption spectrometer equipped with the Glite data station, background correction (deuterium lamp), and appropriate cathode lamps [37]. For Ca determination, a 10% aqueous solution of lanthanum chloride was added to all of the measured solutions in a quantity, ensuring a final La^+3^ concentration of 1%. The determination of the selected elements was performed at the following wavelengths: 324.8 nm (Cu), 279.5 nm (Mn), 248.3 nm (Fe), 213.9 nm (Zn), 285.2 nm (Mg), and 422.7 nm (Ca).

The emission technique (acetylene–air flame) was applied for sodium (Na) and potassium (K) determination with the use of the atomic absorption spectrometer Thermo iCE 3000 Series (Waltham, MA, USA), equipped with a Glite data station, operating in an emission system. Minerals were determined at the following wavelengths: 589.0 nm (Na) and 766.5 nm (K). The content of individual minerals was expressed as the mean of triplicate with standard deviation in 100 g of fresh bread samples.

### 2.6. Folates Analysis

The content of folate vitamers was analyzed as previously described [32]. Briefly, 1 g ± 0.001 g of the blended sample was homogenized in an extraction buffer (0.1 M phosphate buffer, pH 7.0, with 1% (*w*/*v*) sodium ascorbate, and 0.1% (*v*/*v*) 2-mercaptoethanol). After boiling in a water bath for 15 min, samples were cooled in ice. After the addition of α-amylase (Sigma Aldrich) and rat plasma conjugase (Europa Bioproducts Ltd., Cambridge, Great Britain), the samples were incubated at 37 °C for 4 h. After one hour, protease (Sigma Aldrich) was added. Then, they were heated in a boiling water bath for 15 min, cooled in ice, and centrifuged twice at 12,000 rpm/4 °C/20 min. The supernatants were collected, filtered, flushed with nitrogen, and stored at −70 °C. Prior to the high performance liquid chromatography (HPLC) analysis, the samples were purified using solid phase extraction (SPE) on strong anion exchange (SAX) cartridges.

The folate content was determined using a HPLC system (Shimadzu Nexera-i LC-2040 C plus; Shimadzu Co.; Kyoto, Japan) and a C18 LC column (150 × 4.6 mm, 3 µm, Luna 100Å; Phenomenex; Torrance, CA, USA). The total separation time was 42 min, the flow rate was 0.4 mL/min, the injection volume was 20 µL, and the column temperature was 25 °C. Folates were separated under binary gradient elution conditions, with 30 mM phosphoric acid buffer (pH 2.3) and acetonitrile used as the mobile phases. The gradient started at 6% (*v*/*v*) acetonitrile maintained isocratically for the first 5 min, then raised linearly to 25% within 20 min. Quantification of the individual folate vitamers was based on fluorescence detection (290 nm excitation and 360 nm emission) using the external multilevel calibration curves. Peak identification was based on the retention time compared with the standards. The folate content was expressed as means with standard deviations from triplicates per fresh weight (FW) unit. The total folate content was the sum of 5-CH_3_-H_4_folate and H_4_folate expressed as the folic acid content using a molar absorption coefficient given by Blakely [38].

### 2.7. Statistical Analysis

Differences in the mean total folate content and the content of individual minerals in the bread samples were compared using the Duncan multiple range test with a significance level of *P* < 0.05. The data of TPC and antioxidant activity were compared using one-way ANOVA with Fisher’s least significant difference (LSD) post hoc at a significance level of *P* < 0.05. The statistical analysis was carried out using Statistica software version 13.2 (StatSoft; Cracow, Poland).

## 3. Results and Discussion

### 3.1. TPC and Antioxidant Activity Measured by PCL Assay

The results of the total phenolic content (TPC) and antioxidant activity of the breads, controls, and with the incorporation of kale (10 and 20%) and spinach (20 and 40%) are presented in Table 2. The PCL assay was used to measure the ability of hydrophilic and lipophilic antioxidants to scavenge superoxide anion radicals through the ACW and ACL kits, respectively.

The TPC of the toasted and wholemeal bread extracts were determined at the level of 1.33 and 1.73 mg/g, respectively. Therefore, the supplementation significantly increased the phenolic content up to 2–3 times in the bread with the addition of 20% of spinach and 40% of kale. Nazzaro et al. [39] also noticed that kale-based snacks are rich in many health-beneficial components such as polyphenols. Gallic and chlorogenic acids and catechin represent domain secondary metabolites found in snacks with fresh kale incorporation [39]. Furthermore, the TPC in our bread with 10% and 20% of kale (2.00 and 4.10 mg/g) was determined at a higher level than snacks with 56% kale (1.21–3.37 mg/g). This finding might be due to the degradation of polyphenols under the harsh conditions of snack preparation, and thus the extrusion of chips requires a much higher temperature than baking. Furthermore, almost 2- and 3-fold higher values of TPC were determined in bread after the addition of 20 and 40% spinach in comparison to wholemeal bread. The higher TP content in designed baked foods with the addition of spinach could be noticed due to the presence of several domain phytochemicals such as ferulic acid, syringic acid, sinapic acid, and benzoic acid [40]. The ACW values were established at the level of 0.27 in the toasted bread (without kale addition) and 1.32 µmol Trolox/g d.m. in the wholemeal bread (no spinach). Then, the 10% addition of kale increased the antioxidant activity of the bread extracts 12-times, and the 20% amount of kale over 16-times, in comparison to the control toasted bread. Additionally, the 20% incorporation of spinach to bread formulae increased ~2-times the ability of its extract to scavenge O_2_^•−^. Even the breads with the lowest kale and spinach addition achieved significantly higher antioxidant activity than oat-based bread with 80% addition of buckwheat [35], and twice higher than in gluten-free bread with broccoli leaves [33]. Moreover, the 1.8-fold and 2.4-fold increase was noted in the case of bread extracts with 10% and 20% kale, respectively (using ACL protocol), while Krupa-Kozak et al. [33] noticed a significantly higher (about 16%) ACL value for bread with 5% of powdered broccoli leaves than those determined in our study.

According to the definition presented by Lorenzo et al. [41], bioaccessibility is defined as the release of compounds from the food matrix, where they become more available for gastro-intestinal absorption, and several factors (e.g., pH, digestive enzymes, and microbiota) could limit the phenolic bioavailability. Therefore, *in vitro* digestion is implemented to study compound conversions in the gastrointestinal tract conditions and are likely to reach the colon where they can act or be absorbed into the blood stream. Both the TPC and ACW/ACL values increased after the gastric stage when compared to the results without digestion for almost all bread formulations (*P* < 0.05). Bączek et al. [35] reported a 2–3 times higher ACW level when compared to bread extracts. Similar results were published by Lafarga et al. [40], who found that a higher amount of phenolic and antioxidant compounds could be released under the conditions simulating gastrointestinal digestion. However, a decrease in the TPC and antioxidant activity (ACL) was observed for the control breads and the enriched ones after the intestinal stage (*P* < 0.05). The decrease in the antioxidant activity of broccoli-containing crackers after the intestinal stage, using the DPPH (2,2-diphenyl-1-picrylhydrazyl) and FRAP (ferric reducing antioxidant power) methods, was noted [42]. Furthermore, a similar trend was observed in the wild Chilean currants. A decrease in the antioxidant activity was observed throughout the digestion of currant samples, which was correlated with the reduction in the TPC [43]. Only the ACW values increased significantly after intestinal digestion in all bread formulations (*P* < 0.05). Additionally, it is known that antioxidant activity can be affected by the different pH of the medium in which food is dissolved. The structure of polyphenols and their changes depending on pH are responsible for a decrease or increase in the antioxidant activity of these components [44].

### 3.2. Minerals

Fresh spinach and kale are referred as good natural sources of minerals [22,23,45] including potassium (530 mg/100 g and 235 mg/100 g, respectively), magnesium (53 mg/100 g and 30 mg/100 g, respectively), phosphorus (29 mg/100 g and 56 mg/100 g, respectively), calcium (93 mg/100 g and 157 mg/100 g, respectively), iron (2.8 mg/100 g and 1.7 mg/100 g, respectively), zinc (0.7 mg/100 g and 0.44 mg/100 g, respectively), copper (0.10 mg/100 g and 0.09 mg/100 g, respectively), and manganese (0.26 mg/100 g and 0.55 mg/100 g, respectively). However, the chemical composition of vegetables including minerals is very diverse and largely depends on the species and variety, stage of maturity, soil and climatic conditions, harvesting, and storage temperature [46,47]. In turn, the degree of preservation of the mineral elements in vegetables depends on the thermal processing applied, for example, the amount of water used, the degree of raw material grinding, and whether the minerals are in a more or less soluble form. During food processing, minerals may be released from complexes with organic compounds, which may change their biological activity [46]. It is important to know the mineral composition of novel foods as both a deficiency and excess of individual minerals could increase the risk of occurrence of diseases such as osteoporosis, hypertension, cancer, coronary heart disease, and diabetes [25,48].

The characteristics of the selected mineral composition of toasted bread and wholemeal bread with no vegetable addition are presented in Table 3. The obtained results, taking into account the type of bread and flour used, are well in line with the individual mineral amounts reported in the food composition tables [22,23]. The wholemeal bread enrichment with fresh spinach resulted in a significant (*P* < 0.05) increase in all of the analyzed minerals (Table 3). The most spectacular effect was observed for Fe, Mg, Ca and K, where in both breads with the addition of 20% and 40% spinach, the increase exceeded 20% when compared to the individual mineral content in the non-fortified breads. The increase for the 20% and 40% enriched breads was as follows: Fe—22% and 41%; Mg—35% and 48%; Ca—73% and 97%; K—52% and 116%, respectively. In bread with the addition of 40% spinach, a high increase of Cu from 0.17 mg/100 g to 0.23 mg/100 g, 35%, was also noticed. Previous literature data indicate the successful food product enrichment with spinach based powder in terms of mineral content. In the study of Waseem et al. [45], a significant increase (*P* < 0.05) in the levels of K, Ca, and Fe (from 448 to 494 mg/100g, from 40 to 301 mg/100g, and from 3.3 to 11.6 mg/100g, respectively) was reported in chapattis (the staple food in the Indian subcontinent) after wheat flour enrichment with spinach powder (20%). In another study of Galla et al. [19], spinach powder with good quantities of minerals such as calcium 1336 mg/100 g, iron 30 mg/100 g, and phosphorous 336 mg/100 g resulted in a significant increase in minerals in biscuits supplemented with powder.

In the toasted bread enriched with kale (Table 3), a significant (*P* < 0.05) increase was observed for both breads with 10% and 20% vegetable addition for Mn (23% and 61%, respectively), Fe (6% and 19%, respectively), Mg (35% and 67%, respectively), and K (17% and 29%, respectively) compared to non-vegetable breads. The significant (*P* < 0.05) increase in Ca was reported only in bread with the 20% kale addition, from 45.26 mg/100 g to 56.26 mg/100 g, 24%. In turn, both breads with the addition of 10% and 20% kale were characterized by significantly (*P* < 0.05) lower Na (17% and 11%, respectively) and P (9% and 7%) contents compared to the non-enriched bread.

Table 3 also presents the estimated percent coverage of daily demand (DDC%) for the selected minerals calculated based on the RDA (recommended daily allowance) and its content in three slices (100 g) of enriched bread. Depending on the spinach addition, the portion (100 g) of enriched bread could cover from 21% to 26% of daily demand for Cu in the group of adults, and up to 33% in the group of children. Copper is a component of many enzymes involved in the transformation of oxygen and is related to the synthesis of neurotransmitters. It is also essential for iron metabolism and heme synthesis in the body [25]. All tested breads could contribute well to cover the daily Mn requirements in adults—from 17–21% in bread with the lower kale addition up to 37–47% in bread with a higher spinach content. Although manganese deficiencies in humans are rarely described due to, inter alia, its widespread occurrence in food, it plays an important role in the body as a component or activator of enzymes involved in proteins, nucleic acids, and fatty acids [25,49]. In the group of adults, breads enriched with spinach (100 g portion) were demonstrated to cover up to 17% (20% spinach addition) and 19% (40% spinach addition) of the daily demand for Fe, where dietary deficiencies are common and lead to anemia [25]. Breads enriched with spinach contributed better to cover the daily requirements in adults for Zn—even up to 16–22%, Mg—up to 13–18%, and K—12% than breads with the addition of kale. A total of 100 g of both types of enriched breads largely covered the daily requirements for sodium, from 24% to 36% in adults and from 24–34% in children and youth, depending on the kind and amount of added vegetable. In terms of P, the highest possible DDC% was shown for wholemeal breads with 20% and 40% spinach addition of 28% and 29%, respectively, in adults. Meanwhile, excessive phosphorus and sodium amounts in the diet are common [50]. Moreover, too much phosphorus in the diet may adversely affect the absorption of other minerals such as iron, zinc, copper, and magnesium. In turn, an excess of sodium leads to the development of high blood pressure, heart attack, and stroke. It can also significantly increase the incidence of gastric cancer, osteoporosis, and promote the development of obesity [25,50].

### 3.3. Folates

The term folates includes naturally occurring folate derivatives in foods as well as folic acid (FA). FA is the most oxidized folate and therefore, because it has the highest stability to chemical degradation and bioavailability, it is used for supplementation and fortification purposes [51,52,53]. The recommended daily intake of folates depends on age, sex, and physical condition [25,54]. According to the Polish standards*,* the recommended daily allowance (RDA) of folate for adults is 400 µg and is higher, 600 µg, for pregnant and lactating women [25]. Despite a lower folate content than, for example, folate-rich vegetables, bread is reported as one of the most important food contributors to the daily folate intake due to its frequent consumption in various population groups [55]. Taking into account common folate deficiency [28], it is reasonable to enrich bread with natural folates to increase their daily consumption. Our results showed (Table 4) that the major natural folate form determined in the tested samples was 5-CH_3_-H_4_PteGlu (14.5–62.6 µg/100 g) compared to H_4_PteGlu (3.5–20.6 µg/100 g). The higher contribution of the methyl folate form in the tested breads can be explained by the considerable contribution from yeast and vegetables where 5-CH_3_-H_4_PteGlu is the dominant form, as shown in previous studies [10,53,56,57]. The lowest folate content was found in toasted and wholemeal breads with no addition of vegetables, 18.1 µg/100 g and 37.2 µg/100 g, respectively. The folate content results for wholemeal samples with no vegetable addition corresponded well with data available in food composition tables and previous study [10,22,23]. Kunachowicz et al. [22] presented folate content in wheat bread at the level of 30.7 µg/100 g, in wheat “graham” bread—39.0 µg/100 g. Similar values (42 µg/100 g) are provided by the U.S. Department of Agriculture Food Data Central [23] for commercially prepared wheat bread. Higher amounts, compared to our results, were reported for toasted bread, of 44.5 µg/100 g [22]. Studies of other authors [10,57,58,59,60] conducted on wheat and rye breads clearly indicate that the folate content in the final product depends on the types of flour and milling used as well as other ingredients, the most important of which is yeast, as one of the richest natural sources of folate. Hence, properly selected fermentation conditions and the selection of appropriate yeasts may have a beneficial effect on the final folate content in bread [60]. The baking process itself is also important. Significant losses under the influence of temperature and oxygen are widely reported in studies on the effects of various food processing on folate content in different foods [32,52,53,56,61,62,63,64,65] including studies on the bread making process [10,59,60].

In both types of bread, the addition of fresh kale and spinach caused a significant (*P* < 0.05) increase in folates (Table 4). Compared to the non-fortified toasted bread without, the 10% addition of fresh kale resulted in a 53.0% increase in the folate content in bread after baking, while the 20% addition had a 150.3% increase. Additionally, wholemeal breads with the addition of 20% and 40% of fresh spinach showed higher total folate contents of 80.1% and 123.7%, respectively, compared to the non-fortified sample. A positive effect in terms of the folate content of bread fortification with Swiss chard and spinach was presented in an earlier study by López-Nicolás et al. [10]. The authors showed that 20% addition of these green vegetables could result in an approximate 2-fold increase in folate content, and in a 3-fold increase in wheat bread samples with the addition of 40%. The authors also studied the effect of the bread-making process on the folate content, showing that despite a significant decrease in the folate content of raw materials (such as yeast, vegetables and flour) during baking, the fortified breads could contribute well to the recommended daily intake of folate. In our study, the highest contribution to RDA showed wholemeal breads with 20% and 40% spinach addition (Table 4). The 100 g portion of bread (three slices) with the 20% addition of fresh spinach could cover 27% of the daily demand for folate in the group of children, and 17% in adults, and 33% and 21% in the 40% fortified bread, respectively.

Present and previous studies of enriching staple foods with natural folates are of special importance in the situation when other ways of increasing the intake of these vitamins raise doubt. In a previous study [66], we focused on the quality of dietary supplements with folic acid available on the Polish market, indicating that the consumer, due to, among others, the lack of effective control by the relevant sanitary services, is not sure whether the bought product contains the vitamin content declared on the packaging. Other studies indicate that the problem of excessive or too low folic acid addition can also relate to food products voluntarily fortified with folic acid [67,68]. Controversies also surround the mandatory food fortification, most often flour, with synthetic folic acid, introduced in many countries, none of which is European. Although this method has proven to be effective in reducing health problems associated with folate deficiency, in the USA and Canada, a significant reduction in NTD occurrence was observed [69], and some studies have shown possible adverse effects caused by excessive folic acid consumption. These include the occurrence of unmetabolized folic acid in the plasma and pose a health risk (e.g., mask the symptoms of vitamin B12 deficiency or limit the effectiveness of folate antagonists used as drugs in chemotherapy) [70]. Therefore, foods rich in folates of natural origin, which, even when excessively consumed are not associated with the risk of exceeding the upper tolerable level of 1 mg for adults and the occurrence of possible adverse health effects [71], should be promoted in all population groups.

## 4. Conclusions

It has been successfully demonstrated that breads enriched with spinach and kale can be important additional sources of health-promoting nutrients such as folates and individual minerals. In breads with the highest addition of kale (20%) and spinach (40%), the increase in the total folate content was at the level of 150% and 123%, respectively. The tested samples of all variants of enriched breads showed significantly (*P* < 0.05) higher amounts of individual minerals such as Mn, Fe, Mg, and K. A phenolic content up to 66–68% was achieved by the addition of 20% kale and 40% of spinach. Moreover, kale and spinach increased the healthy properties of bread by higher antioxidant status. The highest antioxidant activity was noted in the case of lipid-soluble antioxidants.

The obtained results are promising and encourage further analysis, especially in terms of ingredient selection, the effect of fermentation, and the baking process parameters to obtain a product enriched with bioactive compounds of natural origin at the highest possible level. Importantly, future research must take into account the consumer assessment, so that the product not only has high nutritional value, but also gains the consumers’ acceptability.

## Figures and Tables

**Table 1:** Tested bread formulae.

**Type of Bread**	**Ingredients [g/1000 g]**
Toasted bread	Wheat flour (640), bakers’ yeast (1.5), salt (8), oil (9), sugar (10), milk powder (10)
Toasted bread 10% kale	Wheat flour (590), bakers’ yeast (1.5), salt (8), kale (100), oil (9), sugar (10), milk powder (10)
Toasted bread 20% kale	Wheat flour (550), bakers’ yeast (1.5), salt (8), kale (200), oil (9), sugar (10), milk powder (10)
Wholemeal bread	Wholemeal wheat flour (460), wheat flour (240), bakers’ yeast (1.5), salt (10), oil (9), sugar (10), milk powder (10)
Wholemeal bread 20% spinach	Wholemeal wheat flour (410), wheat flour (215), bakers’ yeast (1.5), salt (10), spinach (200), oil (9), sugar (10), milk powder (10)
Wholemeal bread 40% spinach	Wholemeal wheat flour (310), wheat flour (160), bakers’ yeast (1.5), salt (10), spinach (400), oil (9), sugar (10), milk powder (10)

**Table 2:** The total phenolic content (TPC) and antioxidant activity (extracts (a) and after the *in vitro* digestion (b)) of breads measured by Folin’s reagent (mg/g), and the PCL, ACW, and ACL methods (µmol Trolox/g d.m.).

	PCL [µmol Trolox/g d.m.]		TPC [mg/g]		PCL [µmol Trolox/g d.m.]		TPC [mg/g]
	ACW	ACL			ACW	ACL	
**Extracts**							
Toasted bread	0.27 ± 0.02 ^g^	3.44 ± 0.04 ^h^	1.33 ± 0.08 ^d^	Wholemeal bread	1.32 ± 0.06 ^f^	2.39 ± 0.08 ^g^	1.73 ± 0.17 ^c^
Kale 10%	3.24 ± 0.09 ^e^	6.21 ± 0.06 ^g^	2.00 ± 0.25 ^d^	Spinach 20%	2.50 ± 0.08 ^e^	3.68 ± 0.24 ^fg^	3.25 ± 0.08 ^c^
Kale 20%	4.53 ± 0.08 ^c^	8.21 ± 0.06 ^f^	4.10 ± 0.06 ^d^	Spinach 40%	3.48 ± 0.04 ^d^	4.25 ± 0.22 ^f^	5.15 ± 0.48 ^c^
*In vitro* digestion **‘gastric stage’**							
Toasted bread	1.38 ± 0.22 ^f^	13.34 ± 0.90 ^d^	69.44 ± 5.82 ^a^	Wholemeal bread	5.26 ± 0.60 ^b^	17.00 ± 1.08 ^cd^	63.68 ± 2.00 ^a^
Kale 10%	6.49 ± 0.18 ^b^	10.06 ± 0.17 ^e^	69.62 ± 5.58 ^a^	Spinach 20%	1.22 ± 0.11 ^f^	38.18 ± 2.19 ^b^	65.49 ± 2.83 ^a^
Kale 20%	4.05 ± 0.08 ^d^	21.85 ± 1.17 ^a^	67.44 ± 1.21 ^a^	Spinach 40%	3.54 ± 0.30 ^cd^	40.32 ± 1.34 ^a^	67.15 ± 4.78 ^a^
*In vitro* digestion **‘intestinal stage’**							
Toasted bread	2.51 ± 0.24 ^e^	6.10 ± 0.07 ^g^	53.60 ± 5.91 ^c^	Wholemeal bread	5.83 ± 0.04 ^ab^	8.82 ± 0.06 ^e^	57.39 ± 3.98 ^b^
Kale 10%	5.02 ± 0.33 ^c^	16.81 ± 0.38 ^c^	56.96 ± 4.24 ^bc^	Spinach 20%	4.03 ± 0.27 ^c^	16.89 ± 0.63 ^d^	62.54 ± 0.20 ^ab^
Kale 20%	8.61 ± 0.17 ^a^	19.92 ± 1.19 ^b^	61.81 ± 0.55 ^ab^	Spinach 40%	6.06 ± 0.16 ^a^	18.68 ± 0.28 ^c^	63.70 ± 2.34 ^a^

Data expressed as mean ± standard deviation (*n* = 3); different letters within the same column indicate statistically significant differences at *P* < 0.05 in LSD Fisher test.

**Table 3:** Mineral content in the tested breads and the coverage of the daily demand for selected minerals after consuming a 100 g portion of bread.

Minerals Content ^1^, RDA ^2^/AI ^3^ and DDC ^4^	Toasted Bread		Kale 10%		Kale 20%		Wholemeal Bread		Spinach 20%		Spinach 40%	
	Children and Youth 10–18	Adults ≥19	Children and Youth 10–18	Adults ≥ 19	Children and Youth 10–18	Adults ≥19	Children and Youth 10–18	Adults ≥19	Children and Youth 10–18	Adults ≥19	Children and Youth 10–18	Adults ≥19
Cu [mg/100 g]	0.10 ± 0.01 ^a^		0.09 ± 0.01 ^b^		0.10 ± 0.01 ^a^		0.17 ± 0.01 ^c^		0.19 ± 0.01 ^b^		0.23 ± 0.01 ^a^	
RDA	0.7–0.9	0.9	0.7–0.9	0.9	0.7–0.9	0.9	0.7–0.9	0.9	0.7–0.9	0.9	0.7–0.9	0.9
DDC [%]	11–14	11	10–13	10	11–14	11	19–24	19	21–27	21	26–33	26
Mn [mg/100 g]	0.31 ± 0.01 ^c^		0.38 ± 0.01 ^b^		0.50 ± 0.01 ^a^		0.70 ± 0.01 ^b^		0.82 ± 0.01 ^a^		0.84 ± 0.02 ^a^	
AI	1.5–2.2	1.8–2.3	1.5–2.2	1.8–2.3	1.5–2.2	1.8–2.3	1.5–2.2	1.8–2.3	1.5–2.2	1.8–2.3	1.5–2.2	1.8–2.3
DDC [%]	14–21	13–17	17–25	17–21	23–33	22–28	32–47	30–39	37–55	36–46	38–56	37–47
Fe [mg/100 g]	0.68 ± 0.01 ^c^		0.72 ± 0.02 ^b^		0.81 ± 0.01 ^a^		1.38 ± 0.03 ^c^		1.68 ± 0.02 ^b^		1.94 ± 0.01 ^a^	
RDA	10–15	10–18	10–15	10–18	10–15	10–18	10–15	10–18	10–15	10–18	10–15	10–18
DDC [%]	5–7	4–7	5–7	4–7	5–8	4–8	9–14	8–14	11–17	9–17	13–19	11–19
Zn [mg/100 g]	1.07 ± 0.02 ^a^		0.92 ± 0.01 ^b^		1.05 ± 0.02 ^a^		1.54 ± 0.05 ^c^		1.64 ± 0.03 ^b^		1.73 ± 0.01 ^a^	
RDA	8–11	8–11	8–11	8–11	8–11	8–11	8–11	8–11	8–11	8–11	8–11	8–11
DDC [%]	10–13	10–13	8–12	8–12	10–13	10–13	14–19	14–19	15–21	15–21	16–22	16–22
Mg [mg/100 g]	14.17 ± 0.15 ^c^		19.10 ± 0.16 ^b^		23.61 ± 0.09 ^a^		37.41 ± 0.54 ^c^		50.69 ± 0.55 ^b^		55.45 ± 0.51 ^a^	
RDA	240–410	310–420	240–410	310–420	240–410	310–420	240–410	310–420	240–410	310–420	240–410	310–420
DDC [%]	3–6	3–5	5–8	5–6	6–10	6–8	9–16	9–12	12–21	12–16	14–23	13–18
Ca [mg/100 g]	45.26 ± 1.77 ^b^		45.05 ± 0.37 ^b^		56.26 ± 0.55 ^a^		24.05 ± 0.49 ^c^		41.66 ± 0.33 ^b^		47.33 ± 0.57 ^a^	
RDA	1300	1000–1200	1300	1000–1200	1300	1000–1200	1300	1000–1200	1300	1000–1200	1300	1000–1200
DDC [%]	3	4–5	3	4–5	4	4–5	2	2	3	3–4	4	4–5
Na [mg/100 g]	494.43 ± 4.67^a^		410.16 ± 9.79 ^c^		437.05 ± 11.77 ^b^		319.86 ± 4.42 ^c^		355.25 ± 1.60 ^b^		407.50 ± 7.80 ^a^	
AI	1300–1500	1200–1500	1300–1500	1200–1500	1300–1500	1200–1500	1300–1500	1200–1500	1300–1500	1200–1500	1300–1500	1200–1500
DDC [%]	33–38	33–41	27–32	27–34	29–34	29–36	21–25	21–27	24–27	24–30	27–31	27–34
K [mg/100 g]	161.71 ± 2.25 ^c^		188.58 ± 1.48 ^b^		209.35 ± 0.35 ^a^		193.89 ± 2.45 ^c^		293.87 ± 1.01 ^b^		417.85 ± 4.87 ^a^	
AI	2400–3500	3500	2400–3500	3500	2400–3500	3500	2400–3500	3500	2400–3500	3500	2400–3500	3500
DDC [%]	5–7	5	5–8	5	6–9	6	6–8	6	8–12	8	12–17	12
P [mg/100 g]	112.16 ± 2.58 ^a^		101.57 ± 0.79 ^b^		104.36 ± 3.07 ^b^		186.29 ± 1.91 ^c^		193.44 ± 1.56 ^b^		205.16 ± 4.87 ^a^	
RDA	1250	700	1250	700	1250	700	1250	700	1250	700	1250	700
DDC [%]	9	16	8	15	8	15	15	27	15	28	16	29

^1^ Data expressed as mean ± standard deviation (*n* = 3); different letters within the same row indicate statistically significant difference at *P* < 0.05 in the Duncan test. ^2^ Recommended daily allowance [mg/person/day] based on Jarosz et al. [25]. ^3^ Adequate intake [mg/person/day] based on Jarosz et al. [25]. ^4^ Percent coverage of daily demand for selected minerals calculated based on RDA and its content in the tested bread sample.

**Table 4:** The folate content in the tested breads and the coverage of the daily demand for folates after eating a 100 g portion of bread in various population groups.

				**Group, Age**		
				Children and youth 10–18	Adults ≥19	Pregnant and breast feeding women
**Sample**	**Folates [µg/100 g]**			**RDA ^1^**		
	**H_4_PteGlu**	**5-CH_3_-H_4_PteGlu**	**Total Folates ^2^**	250–400	400	600
				**DDC% ^3^**		
Toasted bread	3.5 ^4^ ± 0.3	14.5 ± 1.3	18.1 ± 1.3 ^a5^	5–7	5	3
Kale 10%	3.7 ± 0.2	24.0 ± 1.4	27.7 ± 1.3 ^b^	7–11	7	5
Kale 20%	4.8 ± 0.2	40.5 ± 2.6	45.3 ± 2.4 ^c^	11–18	11	8
Wholemeal bread	10.1 ± 0.9	27.1 ± 2.2	37.2 ± 2.5 ^a^	9–15	9	6
Spinach 20%	13.8 ± 1.3	53.3 ± 4.6	67.0 ± 4.8 ^b^	17–27	17	11
Spinach 40%	20.6 ± 1.0	62.6 ± 4.9	83.2 ± 4.7 ^c^	21–33	21	14

^1^ Recommended daily allowance for folates [µg folate/person/day] based on Jarosz et al. [25]. ^2^ Total folates is the sum of H_4_ folate and 5-CH_3_-H_4_ folate expressed as the equivalent of folic acid. ^3^ Percent coverage of daily demand for folates calculated based on RDA and the total folate content in the tested bread sample. ^4^ Data expressed as mean ± standard deviation (*n* = 3); ^5^ Different letters within the same column indicate statistically significant difference at *P* < 0.05 in the Duncan test.

## Data Availability

The data are available from the corresponding author.
